# The economic impact of anastomotic leak after colorectal cancer surgery

**DOI:** 10.1186/s13561-023-00425-y

**Published:** 2023-02-16

**Authors:** Blas Flor-Lorente, José Francisco Noguera-Aguilar, Salvadora Delgado-Rivilla, José María García-González, Marcos Rodriguez-Martín, Laura Salinas-Ortega, Miguel Ángel Casado, María Álvarez

**Affiliations:** 1 Colorectal Surgery Unit. Hospital Universitario y Politécnico La Fe, Valencia, Spain; 2 Colorectal Surgery Unit. Complejo Hospitalario y Universitario A Coruña, A Coruña, Spain; 3 Colorectal Surgery Unit, Hospital Universitario Mutua de Tarrassa, Barcelona, Spain; 4 Colorectal Surgery Unit, Hospital Universitario Cruces, Bilbao, Spain; 5 Colorectal Surgery Unit, Hospital Beata María Ana, Madrid, Spain; 6 Pharmacoeconomics & Outcomes Research Iberia (PORIB), Madrid, Spain; 7 Health Economics & Outcomes Research Unit (Medtronic Ibérica, S.A.), Madrid, Spain

**Keywords:** Anastomotic leak, Colorectal cancer, Cost, Resection, Stoma, Spain

## Abstract

**Objective:**

To determine the economic impact of the incremental consumption of resources for the diagnosis and treatment of anastomotic leak (AL) in patients after resection with anastomosis for colorectal cancer compared to patients without AL on the Spanish health system.

**Method:**

This study included a literature review with parameters validated by experts and the development of a cost analysis model to estimate the incremental resource consumption of patients with AL versus those without. The patients were divided into three groups: 1) colon cancer (CC) with resection, anastomosis and AL; 2) rectal cancer (RC) with resection, anastomosis without protective stoma and AL; and 3) RC with resection, anastomosis with protective stoma and AL.

**Results:**

The average total incremental cost per patient was €38,819 and €32,599 for CC and RC, respectively. The cost of AL diagnosis per patient was €1018 (CC) and €1030 (RC). The cost of AL treatment per patient in Group 1 ranged from €13,753 (type B) to €44,985 (type C + stoma), that in Group 2 ranged from €7348 (type A) to €44,398 (type C + stoma), and that in Group 3 ranged from €6197 (type A) to €34,414 (type C). Hospital stays represented the highest cost for all groups. In RC, protective stoma was found to minimize the economic consequences of AL.

**Conclusions:**

The appearance of AL generates a considerable increase in the consumption of health resources, mainly due to an increase in hospital stays. The more complex the AL, the higher the cost associated with its treatment.

**Interest of the study:**

it is the first cost-analysis study of AL after CR surgery based on prospective, observational and multicenter studies, with a clear, accepted and uniform definition of AL and estimated over a period of 30 days.

**Supplementary Information:**

The online version contains supplementary material available at 10.1186/s13561-023-00425-y.

## Introduction

Cancer is one of the diseases with the greatest impact on public health and is the second leading cause of death in Europe [1]. In Spain, colorectal cancer (CRC) is frequently diagnosed [2], mostly in people older than 50 years, with an average age of onset between 70 and 71 years [3] and with a higher incidence in men than in women [4]. The therapeutic approach depends on the type of tumour, whether it has metastasized and the functional status of the patient [5]. Even today, the treatment of choice for colorectal carcinoma is surgery with the objective of removing the primary tumour and its locoregional extension [6].

One of the complications with the greatest clinical repercussions after colorectal cancer surgery is anastomotic leak (AL), defined as a defect of the intestinal wall at the site of the anastomosis, which involves communication between the intra- and extraluminal space [7]. Although its incidence and time of appearance are variable, most ALs appear during the first 2 weeks after anastomosis, although there are cases that occur later [8–10]. Given the great variability of the incidence data due to the multiple definitions of AL, several classifications have been proposed based on the definition of AL, time of appearance, management or degree of complexity. The most accepted classification is that proposed by Rahbari et al. [7], which consists of three types of AL based on their management: type A AL, anastomotic loss that does not require active therapeutic intervention (accidental finding in a routine imaging test or for other reasons); type B AL, which requires active therapeutic intervention but is manageable without surgery; and type C AL, which requires surgical intervention.

Due to the high incidence of CRC and the proportion of patients who undergo resection surgery, two multicentre and prospective studies have been conducted at the national level to determine the risk factors related to AL and the real incidence rates in patients with colon cancer (CC) [11] and rectal cancer (RC) in Spain [10].

Patients with AL require greater postoperative follow-up, since they may require one or more surgical interventions, be admitted to an intensive care unit (ICU), or even require a stoma [12]. This generates longer stays in the hospital and a greater consumption of health resources, which is associated with an increase in health care costs [13]. In this sense, cost studies allow quantifying and assessing in monetary units the total effects of a given disease or pathological condition to estimate the financial impact of the burden of this disease [14].

The average cost generated by patients with CRC, depending upon the cancer stage, has been estimated at between €8813 (in situ stage) and €49,518 (advanced stage) including all the costs generated from the treatment of the disease for 1 year. The cost of the interventions and the hospital stay are the greatest expenses, accounting for 55.2 and 72.0% of the total, respectively [15].

The present analysis examines the economic impact of CRC complications on the health system in Spain. Specifically, we estimated the incremental economic burden for the diagnosis and treatment of AL in adult patients following a resection with anastomosis due to CRC with regards to patients who do not develop ALs.

## Methods

For the development of the analysis, a panel of experts devised parameters for a literature review, which was then conducted. Next, a cost analysis model was developed to estimate the economic impact of the increased consumption of health resources for the diagnosis and treatment of patients with AL compared to CRC patients without this complication.

### Literature review

The literature review was performed with a structured search of the electronic database Medline (PubMed), scientific societies and associations, such as the Spanish Society of Medical Oncology (SEOM, for its Spanish acronym), Spanish Association of Coloproctology (AECP, for its Spanish acronym) and the Spanish Association of Surgeons (AEC, for its Spanish acronym). In addition, a grey literature search was carried out to obtain all reports outside of the databases consulted. The search strategy in PubMed was performed using the following terms: “cost”, “anastomotic leak”, “resection”, “cancer”, “colon”, “rectal” and “colorectal”. This search was limited to articles published in English or Spanish with no limit on the year of publication. To obtain data on the epidemiology of CRCs in Spain, the incidence of AL and its classification according to the treatment of the patient, articles with data referring to Spain were prioritized [10, 11].

### Panel of experts

For the validation of the parameters and the conceptualization of the analysis, several online and face-to-face sessions were held with a panel of five experts in colorectal surgery (BFL, JFNA, SDR, JMGG, and MRM), who are members of scientific committees or boards of the AECP and the AEC.

The panel validated epidemiological data and the cost estimates for the diagnosis and treatment of AL.

### Definitions

#### Anastomotic leak (AL)

Defect of the integrity of the intestinal wall at the colorectal or coloanal anastomotic site (including the suture and staple lines of neorectal reservoirs) that leads to communication between the intra- and extraluminal compartments. A pelvic abscess near the anastomosis is also considered an anastomotic leak, according to the International Study Group of Rectal Cancer (ISREC) [16]. In addition, in our study, all ALs that occurred during the first 30 days after surgery were considered and were classified according to Rahbari et al. [7] as one of the following:

#### AL type A

Does not require an active therapeutic intervention. Normally, type A is detected by routine radiological examinations since the appearance of clinical symptoms or alteration of analytical parameters is not common [7]. Therefore, this classification would only be applicable in RC surgery with routine postoperative radiological tests.

#### AL type B

This type can present clinical symptoms such as distress or abdominal or pelvic pain, and it can even produce air, purulent or faecal material through the drainage, wound or rectum. In addition, these patients usually present with leukocytosis and increased C-reactive protein (CRP). Therefore, they require active therapeutic intervention (percutaneous drainage or antibiotic treatment) without the need for a second surgery [7].

#### AL type C

Requires urgent reoperation since it generally presents purulent or faecal drainage and/or clinical symptoms of peritonitis and/or laboratory signs of infection [7].

### Design of the cost analysis model

**Fig. 1:**
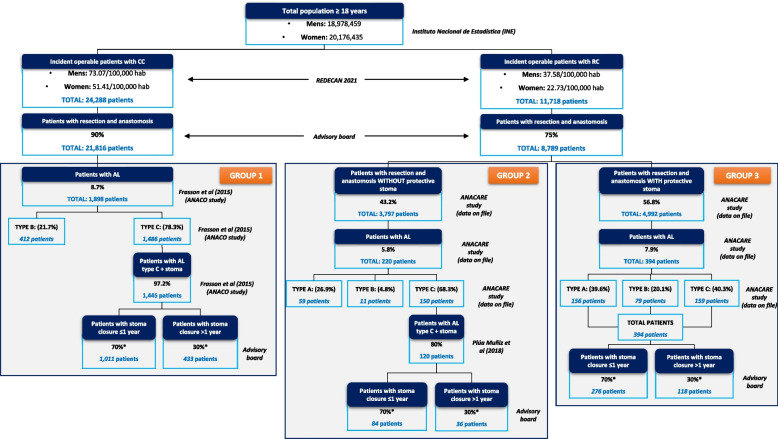
Flow of patients after resection with anastomosis due to colorectal cancer in Spain. *The difference between patients with stoma closure ≤1 year vs. stoma closure > 1 year only impacts the cost of stoma closure; AL: anastomotic leak; CC: colon cancer; RC: rectal cancer

A model was developed using the Microsoft Excel programme to estimate the costs generated for the diagnosis and treatment of AL in adult patients with CRC (Fig. 1). The patients were divided into three main groups according to the pathology and the treatment performed:

- GROUP 1: patients with resection and anastomosis due to CC and suffering from AL.
- GROUP 2: patients with resection and anastomosis due to RC, without protective stoma and experiencing AL.
- GROUP 3: patients with resection and anastomosis due to RC, with protective stoma and experiencing AL.

In the cost estimations for the diagnosis and treatment of AL, only those resources that are modified due to AL have been considered. Therefore, GROUPS 1, 2 and 3 reflect the increased costs incurred when CRC patients experience AL, as determined by the Spanish National Health System.

### Population of study

For the estimation of costs, we considered adult patients with CRC who underwent resection of the colon or rectum with anastomosis and developed AL. Based on the total Spanish adult population, epidemiological data and clinical management were applied to obtain statistics on CRC patients (Fig. 1).

The population of Spain, as determined by the National Institute of Statistics (INE, for its Spanish acronym), reported that the adult population (≥18 years) residing in Spain on January 1, 2021, was 39,154,892, of whom 20,176,435 were women and 18,978,459 were men [17]. The incidence rates of CC and RC were obtained from data published by the Spanish Network of Cancer Registries (REDECAN, for its Spanish acronym) for the year 2021 [18]. To the numbers of CC and RC cases, data on the proportion of patients with anastomosis resection surgery by tumour type were applied. The incidence rates, proportions of AL and rates of patients with a definitive stoma due to AL are based on a 2015 report from the ANACO study group [11], which included 3193 patients with CC and on a report including 1832 patients with RC (National Registry on Leaks in Anastomosis after Surgery for Rectal Cancer) (ANACARE study) [10, 19].

### Time horizon and perspective

The time horizon established for the cost analysis was 1 year after the first resection with anastomosis due to CRC.

The perspective established for the analysis was that of the Spanish National Health System.

### Resources and costs

In accordance with the perspective chosen for the analysis, only direct health costs were considered (i.e., expenses derived directly from the treatment of AL in the healthcare field, such as consultations, follow-up, diagnostic tests, interventions, stoma closure, drugs and any other health resources). Further, only those resources that were increased as a result of the occurrence of AL were identified and quantified (see Additional file 1). Subsequently, unit costs were applied to each resource from a database of health costs in Spain (eSalud) [20] (see Additional file 2).

In the case of patients who required a period of hospitalization either for admission to an ICU or for admission to the ward, the days were calculated for each of the groups of patients with AL.

All costs were expressed in 2021 euros.

## Results

Based on the selected epidemiological data, it was estimated that the number of patients with operable CC and RC in 2021 was 24,288 and 11,718, respectively. A total of 90.0% of patients with CC and 75.0% with RC were candidates for a resection with anastomosis for the removal of tumours. Thus, 21,816 patients with CC and 8789 patients with RC would undergo intestinal anastomosis and might develop AL (Fig. 1).

In the case of patients in GROUP 1, and according to the data obtained from the ANACO study [11], the overall rate of AL was 8.7% (no (0.0%) type A AL patients, 412 (21.7%) type B AL patients and 1486 (78.3%) type C AL patients). In addition, 97.2% of patients who have a second surgery for type C AL undergo a stoma, which is reconstructed during the first year in 70.0% of cases (Fig. 1).

In patients with RC, and according to the data of the ANACARE study [10], 56.8% of patients underwent a protective stoma after resection with anastomosis. In the group without stoma (GROUP 2), the overall rate of AL was 5.8% (59 (26.9%) type A AL patients, 11 (4.8%) type B AL patients and 150 (68, 3%) type C AL patients). In patients with protective stoma (GROUP 3), the rate of AL was 7.9% (156 (39.6%) type A AL patients, 79 (20.1%) type B AL patients) and 159 (40.3%) type C AL patients) (Fig. 1).

The incremental total annual cost of AL was €84,151,002 for CC (GROUP 1) and €20,769,368 for RC (GROUPS 2 and 3). The average per patient with CC was €38,819 and €32,599 for patients with RC. Among patients with RC, the average cost for patients without protective stoma was €34,704 compared to €29,792 for patients with stoma, (22.8% reduction).

The cost of AL diagnosis for CC (GROUP 1) was €1,932,982 and €633,050 for RC (GROUPS 2 and 3) (Table 1), representing 2.3% of the costs for patients with CC (€1018 per patient) and 3.0% in RC (€1030 per patient). The cost of AL treatment for CC (GROUP 1) was €82,218,021, and for RC (GROUPS 2 and 3) €20,136,318, representing 97.7% of costs for patients with CC (€37,800) and 97.0% (€31,569) for patients with RC. According to the classification of the treatment of AL [7], in the CC (GROUP 1), the costs per patient ranged from €18,996 (type B AL) to €50,228 (type C AL + stoma). In the RC (GROUP 2 and 3), the costs per patient ranged from €13,988 (type A AL without protective stoma) to €51,037 (type C AL + stoma).

**Table 1:** Total costs of diagnosis and treatment of AL per patient and for GROUPS 1–3

**AL diagnosis**	**Patients**	**GROUP 1 (patients with CC)**			**GROUP 2 and GROUP 3 (patients with RC)**						
		**1.898**			**615**						
	**Costs (€)**										
	***Consultations***^***a***^	€472			€472						
	***Diagnostic test***^***b***^	€547			€559						
	**TOTAL COST PER PATIENT**	**€1018**			**€1030**						
	TOTAL DIAGNOSTIC COST*	**€1,932,982**			**€633,050**						
**AL treatment**	**Patients**	**GROUP 1 (patients with CC)**			**GROUP 2 (patients with RC without protective stoma)**				**GROUP 3 (RC patients with protective stoma)**		
		**AL type B**	**AL type C without stoma**	**AL type C + stoma**	**AL type A**	**AL type B**	**AL type C without stoma**	**AL type C + stoma**	AL type A **	AL type B**	AL type C**
		**412**	**42**	**1.445**	**59**	**11**	**30**	**120**	**156**	**79**	**159**
	**Costs (€)**										
	***Hospital stay***^***c***^	€9656	€17,708	€17,708	€9966	€13,834	€18,239	€18,239	€9303	€12,640	€16,874
	***Consultation***^***d***^	€2596	€4992	€7605	€1834	€3463	€5098	€7711	€3088	€4663	€6454
	***Test***^***e***^	€2566	€3428	€3649	€1955	€2508	€3254	€3782	€2088	€3171	€3916
	***Interventions***^***f***^	–	€9668	€11,602	–	–	€9668	€11,602	–	–	€7735
	***Stoma closure***	–	–	€1181	–	–	–	€1181	€1378	€984	€984
	***Drugs***^***g***^	€2354	€3139	€3139	–	€3139	€3139	€3139	–	€3139	€3139
	***Other recourses***^***h***^	€1825	€5242	€5344	€232	€2088	€5242	€5383	€334	€1877	€4518
	**TOTAL COST PER PATIENT**	**€18,996**	**€44,177**	**€50,228**	**€13,988**	**€25,032**	**€44,639**	**€51,037**	**€16,191**	**€26,474**	**€43,620**
	TOTAL TREATMENT COST*	**€7,823,779**	**€1,838,298**	**€72,044,183**	**€828,573**	**€264,588**	**€1,342,767**	**€6,098,265**	**€2,463,916**	**€2,075,118**	**€6,885,594**

*AL* Anastomotic leak, *CC* Colon cancer, *RC* Rectal cancer; * For all patients in each group and type of AL. **In the patients of GROUP 3, there is a decrease in the cost generated for the closure of the stoma since a percentage of the patients do not have the closure of the stoma in the first year as a consequence of AL

^a^ Surgeon consultation + radiologist consultation + emergency visit

^b^ Cost of CRP test + procalcitonin + blood test + CT + CT enema

^c^ Cost of inpatient stay + ICU + emergencies

^d^ Surgeon consultation + Radiologist consultation + Stomachtherapist consultation + Nutritionist/internist/rehabilitator consultation + post-operative visits (inpatient)

^e^ Blood test + Colonoscopy + C-reactive protein (CRP) + procalcitonin + Rectoscopy + CT + CT enema

^f^ Reintervention (see Additional file 2)

^g^ Cost of antibiotics and their administration

^h^ Percutaneous drainage + transanal drainage + stoma material + enteral nutrition + parenteral nutrition

**Fig. 2:**
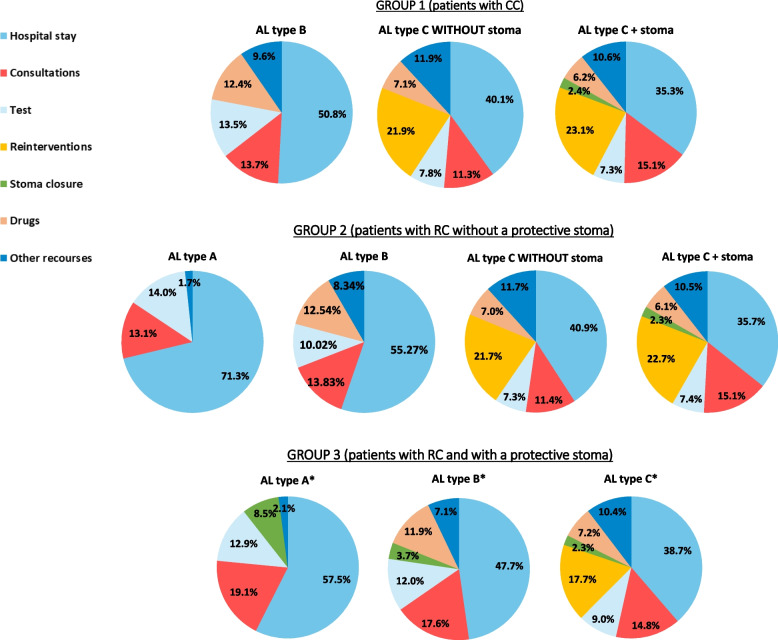
Proportion of costs (€, 2021) of AL treatment in patients with colorectal cancer. *In GROUP 3 patients, there is a decrease in the cost generated for stoma closure since a percentage of patients do not undergo stoma closure in the first year as a result of AL

Regarding the distribution of the AL treatment costs, the hospital stay represents the highest cost item in all groups, ranging from 35.3% for patients in GROUP 1 with type C AL + stoma to 71.3% in patients in GROUP 2 with type A AL. The second highest cost was associated with surgical reoperations in patients with type C AL regardless of the type of neoplasia and in patients with type A/B AL (Fig. 2).

**Fig. 3:**
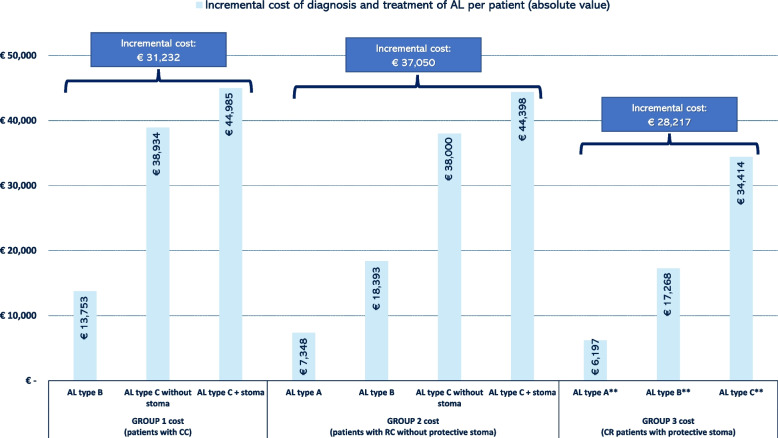
Incremental total costs per patient* (€, 2021) of patients with vs. without AL. *In the estimation of the incremental cost of diagnosis and treatment of AL, only those resources that are modified due to the appearance of AL have been considered. In no case is it intended to capture the total cost associated with the treatment of a patient with colorectal cancer who does not have AL. Therefore, GROUPS 1, 2 and 3 reflect the incremental cost incurred by the National Health System after the onset of AL.**In GROUP 3 patients, there is a decrease in the cost generated for closure of the stoma since a percentage of patients do not have the stoma closed in the first year as a consequence of AL

When comparing the costs generated by a patient with AL vs. without AL (Fig. 3), in GROUP 1, the costs varied from €13,753 (AL type B) to €44,985 (AL type C + stoma), assuming an average increase of more than €30,000. In GROUP 2, costs varied from €7348 (AL type A) to €44,398 (AL type C + stoma), assuming an average increase of more than €35,000. For GROUP 3, the costs varied from €6197 (AL type A) to €34,414 (AL type C), assuming an average increase of slightly less than €30,000. In patients with RC, the costs per patient were substantially lower in those patients with a stoma (GROUP 3) compared to those without a protective stoma (GROUP 2), specifically 16% (AL type A), 6% (AL type B) and 58% (AL type C).

## Discussion

The present analysis has estimated the incremental economic impact of AL complications in patients with resection and anastomosis due to CRC, demonstrating a very considerable increase in the patients’ healthcare cost burden in addition to the physical and psychological burden they bear [21, 22]. Hence, it is very important to do everything possible to prevent AL.

Despite the high incidence of CRC and the high rates of AL, the literature on the economic burden generated by this complication is scarce and focuses on the costs related to the surgical interventions [23], short hospital stays [23, 24], or patients not exclusively oncological [23–25]. This lack of evidence highlights the need to provide surgeons and medical facility managers with useful data to become aware of the clinical and economic magnitude of the problem and to take action to prevent AL. In this sense, our study is the first to estimate the annual cost of AL management in patients with CRC using two prospective, multicentre audits conducted at the national level [10, 11]. This is unlike the literature, which has depended on data obtained from groups related to diagnosis (DRGs), retrospective studies or databases of single hospitals [23–26].

The appearance of AL entails a high consumption of health resources, even in the mildest form, such as AL type A, with respect to patients without AL [15]. Half or even more than half of the patients with AL will undergo surgical reinterventions and the other half will require medical or radiological treatment that will lengthen the hospital stay, representing a very significant increase in costs, that, as per our study, ranges from €6197 per patient with type A AL in GROUP 3 to €44,985 per patient with type C AL and stoma in GROUP 1. As our study has shown, the greater the complication of AL is, the higher the cost. These complications not only generate a greater health expenditure but also significantly affect the patients’ quality of life [27, 28], since the stomas performed in this type of intervention often become definitive [29].

Regardless of the type of neoplasm (CC or RC) and type of AL (A, B or C), the hospital stay (inpatient stay, ICU and emergency room visits) represents the single greatest expense in the diagnosis and treatment of AL, a conclusion similar to that of other studies [25]. In patients with RC who have type A AL, the overall cost is mainly due to the prolonged hospital stay. However, in patients with type C, the cost of the hospital stay is not as significant as the cost of reinterventions that account for 21.3% of the total cost of AL, due to a second surgery.

In a 2021 retrospective, single-centre observational study, Capolupo et al. [25] examined the data of 317 patients who underwent a colon or rectal resection with anastomosis, not necessarily because of a colorectal neoplasia. The incidence rate of AL was 12.3%, 5.9% (14/237) in patients with colon resection and 31.3% (25/80) in patients with rectal resection, much higher than the 8.2% reported by our study (8.7% CC and 7.0% RC). The average cost per patient admitted for the treatment of AL was €14,782, without differentiation by the type of AL or pathology, 108% higher than patients without AL. In our study, the average cost for the treatment of AL per patient was much higher (€33,438 on average, €37,800 for CC and €31,569 for RC), which represents an increase of 128%. However, in our study, expenses per patient per year were considered, not only for the hospital episode, and focused exclusively on patients with CRC.

A study by Hammond et al. [24] in the USA, based on the Premier Perspective™ database (Premier, Inc., Charlotte, NC, USA), examined data from different hospitals retrospectively collected from January 2008 to December 2010. Of the 99,879 patients who underwent colorectal surgery, 6.18% had AL (clinical AL 30 days after the intervention), a rate slightly lower than that observed in our study (8.2%). The additional cost per patient with AL was $24,129 (€21,260, in 2014), with hospital stays similar to those estimated in our analysis (26.3 [24] vs. 26 days). These costs are lower than those reported in our study (€33,438 on average; €37,800 for CC and €31,569 for RC), where a one-year time horizon after surgery was considered.

As demonstrated in multiple studies, performing a protective stoma minimizes the consequences of AL and even decreases the rate of reoperations but not its occurrence [30]. In our study, it is demonstrated that having a protective stoma also minimizes AL costs. When comparing the additional costs of AL in patients without a protective stoma (GROUP 2) versus patients with a protective stoma (GROUP 3), the increased costs per patient were €37,050 and €28,217, respectively. These results are according to Koperna et al. [31], who estimated the cost of significant leakage increased fivefold (from €8400€ to €42,250€) in patients without protective stoma after colorectal anastomosis.

The hospital costs obtained are similar to those reported by the observational study conducted by Ashraf et al. [32] in the UK with 285 patients and a 10.9% AL rate. They found the additional cost per hospital episode for patients with AL was up to £10,901 (€13,065 in 2009). In our study, the average cost of hospitalization for AL treatment was €14,417. In addition, conclusions similar to ours were obtained with respect to the difference in costs for patients with and without protective stoma, agreeing that patients with protective stoma experience a 22.6% reduction in hospital costs, similar to our 22.8% reduction. This is yet another reason, in addition to clinical ones, to perform a protective stoma in at-risk colorectal anastomoses, despite the fact that ileostomies are not free of complications and further costs related to the ileostomy and its subsequent closure [23].

In other retrospective publication, such as the one carried out in the USA by Lee et al. [23], it is estimated that the average patient with AL pays $30,670 (€27,024 in 2015) more than the average patient without AL (1.88 times greater), which represents a 167.5% greater cost (in our study, a 152% average increase). These costs, mainly due to prolonged hospital stays, can increase up to 513% per patient, according to Riberio et al. [26].

The comparison of the economic impact of AL referred to in the publications discussed above is very complex, due to differences in the definition of AL and the methodology of estimation and evaluation of costs. Standardization in the definition of AL is fundamental in this process [32], hence the importance of our study, in which the data source for cost calculation come from multicentric and national audits with a clearly established definition of AL. However, all published studies conclude that the economic impact of AL is very high, regardless of the context or country of interest. It can thus be assumed that AL prevention may result in cost savings and a reduction of clinical burden, leading to a more rationalized use of hospital resources, and potentially a reinforced focus on training.

Our analysis is not without limitations. To obtain data related to patient flow, literature from various sources was used. In addition, it was necessary to make some assumptions to determine health resources and hospital stay in patients with AL due to the absence of data in the literature. In any case, these were validated by the expert panel based on their experience. Another limitation is the possible overestimation of costs due to the adjustment of patients with CC (GROUP 1) according to the Rahbari et al. [7] classification, which was designed for RC. In addition, we have only considered the direct healthcare costs during the year after the first resection with anastomosis due to a CRC, without taking into account costs associated with the patient’s rehabilitation program or costs derived from the maintenance of the stoma. Further, costs, such as lost time at work, have not been included, which makes the overall costs of AL even higher.

## Conclusions

After resection surgery with anastomosis in patients with CRC, the appearance of AL implies an increase in the consumption of health resources compared to patients without AL. Likewise, the greater the complexity of AL, the greater the cost associated with its treatment. In addition, AL can lead to other complications, generating even higher costs. The resources that are increased in greater amounts are hospital stays, and in type C AL, the cost of interventions and stoma closure is added**.** Protective ileostomy in patients with RC minimizes the clinical consequences of AL and, consequently, the economic costs.

These conclusions are of great importance for surgeons and health managers to include AL in internal audits that validate good work in the services or units of colorectal surgery and dedicate more resources to the training of surgeons and the technological development that reduces the incidence of anastomotic leaks.

## Supplementary Information

**Additional file 1: Table S1.** Consumption of resources for the diagnosis of anastomotic leak (AL). **Table S2.** Consumption of resources for the treatment of AL in GROUP 1 (patients with CC and AL) vs. patients without AL. **Table S3.** Consumption of resources for the treatment of AL in GROUP 2 (patients with RC and AL) vs. patients without AL. **Table S4.** Consumption of resources for the treatment of AL in GROUP 3 (patients with RC and with a protective stoma suffering from AL) vs. patients without AL.

**Additional file 2: Table S5.** Unit cost of resources for the diagnosis and treatment of AL.

## Data Availability

All data generated or analysed during this study are included in this published article and its supplementary information files.

## Abbreviations

AL: Anastomotic leak
CC: Colon cancer
CRC: Colorectal cancer
DRGs: Groups related to diagnosis
ICU: intensive care unit
RC: Rectal cancer
CT: Computed tomography

## References

[1] Badía X, Tort M, Manganelli A-G, Camps C, Díaz-Rubio E (2019). The burden of cancer in Spain. Clin Transl Oncol.

[2] Las cifras del cáncer en España (2021). Sociedad Española de Oncología Médica (SEOM).

[3] González Flores E (2020). Cáncer de colon y recto.

[4] Diagnóstico y prevención del cáncer colorrectal. Asociación Española de Gasteroenterologíua y Sociedad Española de Medicina de Familia y Comunitaria. Madrid; 2018 [cited 2021 Jul 1]. Available from: https://www.semfyc.es/wp-content/uploads/2019/01/Actualizacion_Prevencion_cancer_colorrectal_Semfyc.pdf

[5] Cajaraville G, Carreras MJ, Massó J, Tamés MJ (2002). Oncología.

[6] Ayuso ML, Grávalos CC (2007). Guía Clínica Diagnóstico y Tratamiento del Carcinoma Colorrectal.

[7] Rahbari NN, Weitz J, Hohenberger W, Heald RJ, Moran B, Ulrich A (2010). Definition and grading of anastomotic leakage following anterior resection of the rectum: a proposal by the international study Group of Rectal Cancer. Surgery.

[8] Placer C, Vega J, Aguirre I, Rose S, Saralegui Y, Enríquez-Navascués JM (2019). Late anastomotic leakages in rectal surgery: a wake-up call about their impact on long-term results. Cir Cir.

[9] Hyman N, Manchester TL, Osler T, Burns B, Cataldo PA (2007). Anastomotic leaks after intestinal anastomosis: it’s later than you think. Ann Surg.

[10] Protocolo del Registro Nacional sobre fugas en ANAstomosis tras cirugía de CAncer de REcto (ANACARE) Asociación Española de Cirujanos; 2016. 30. Available from: https://www.aecirujanos.es/files/miniwebs/menus/81/documentos/ANACARE_PROTOCOLO.pdf

[11] Frasson M, Flor-Lorente B, Rodríguez JLR, Granero-Castro P, Hervás D, Alvarez Rico MA (2015). Risk factors for anastomotic leak after Colon resection for Cancer: multivariate analysis and nomogram from a multicentric, prospective, National Study with 3193 patients. Ann Surg.

[12] Ais G, Fadrique B, Monge N, Etreros J, Hernández S (2020). Estomas de protección en la cirugía del cáncer de recto: ni siempre ileostomía ni siempre colostomía; adaptándose al paciente. Rev Acircal.

[13] Frye J, Bokey EL, Chapuis PH, Sinclair G, Dent OF (2009). Anastomotic leakage after resection of colorectal cancer generates prodigious use of hospital resources. Color Dis.

[14] Soto Álvarez J. Estudios de farmacoeconomía: ¿por qué, cómo, cuándo y para qué? Medifam. 2001;11. Available from: http://scielo.isciii.es/scielo.php?script=sci_arttext&pid=S1131-57682001000300004&lng=en&nrm=iso&tlng=en. Cited 2022 Apr 8

[15] Corral J, Borràs JM, Chiarello P, García-Alzorriz E, Macià F, Reig A (2015). Estimación del coste hospitalario del cáncer colorrectal en Cataluña. Gaceta Sanitaria. Sociedad Española de Salud Pública y Administración Sanitaria (SESPAS).

[16] Kulu Y, Ulrich A, Bruckner T, Contin P, Welsch T, Rahbari NN (2013). Validation of the international study Group of Rectal Cancer definition and severity grading of anastomotic leakage. Surgery.

[17] Statistics National Institute (INE) (2021). Cifras de población y Censos demográficos-Últimos datos.

[18] Red Española de Registros de Cáncer (REDECAN) (2021). Estimación de la incidencia de cáncer en España.

[19] Flor-Lorente B. Results of the Registro Nacional sobre fugas en ANAStomosis tras cirugía de CÁncer de REcto (ANACARE) presented in the XXIII National Foundation Congress of Asociación Española de Coloproctología (RNAECP 2019) in May 15-17, 2019, Valladolid, Spain. Valladolid; 2019. Available from: https://rnaecp2019.com/index.php/comunicaciones. Cited 2020 Oct 13

[20] Oblikue - Base de conocimiento de costes y precios del sector sanitario. Available from: http://esalud.oblikue.com/. Cited 2021 Jul 1

[21] de Lacy FB, Turrado-Rodriguez V, Torroella A, van Laarhoven J, Otero-Piñeiro A, Almenara R (2022). Functional outcomes and quality of life after Transanal Total Mesorectal excision for rectal Cancer: a prospective observational study. Dis Colon Rectum.

[22] Kverneng Hultberg D, Svensson J, Jutesten H, Rutegård J, Matthiessen P, Lydrup M-L (2020). The impact of anastomotic leakage on long-term function after anterior resection for rectal Cancer. Dis Colon Rectum.

[23] Lee SW, Gregory D, Cool CL (2020). Clinical and economic burden of colorectal and bariatric anastomotic leaks. Surg Endosc.

[24] Hammond J, Lim S, Wan Y, Gao X, Patkar A (2014). The burden of gastrointestinal anastomotic leaks: an evaluation of clinical and economic outcomes. J Gastrointest Surg.

[25] Capolupo GT, Galvain T, Paragò V, Tong C, Mascianà G, Di Berardino S (2022). In-hospital economic burden of anastomotic leakage after colorectal anastomosis surgery: a real-world cost analysis in Italy. Expert Rev Pharmacoecon Outcomes Res..

[26] Ribeiro U, Tayar DO, Ribeiro RA, Andrade P, Junqueira SM (2019). The clinical and economic burden of colorectal anastomotic leaks: middle-income country perspective. Gastroenterol Res Pract.

[27] Calderón C, Jiménez-Fonseca P, Hernández R, Mar Muñoz MD, Mut M, Mangas-Izquierdo M (2019). Quality of life, coping, and psychological and physical symptoms after surgery for non-metastatic digestive tract cancer. Surg Oncol.

[28] De Frutos MR, Solís Muñoz M, Hernando López A, Béjar Martínez P, Navarro Antón C, Mayo Serrano N (2011). Calidad de vida de los pacientes con colostomía e ileostomía a corto y medio plazo. Metas de Enfermería.

[29] Zhang B, Zhuo G-Z, Zhao K, Zhao Y, Gao D-W, Zhu J (2022). Cumulative incidence and risk factors of permanent stoma after Intersphincteric resection for ultralow rectal Cancer. Dis Colon Rectum.

[30] Tan WS, Tang CL, Shi L, Eu KW (2009). Meta-analysis of defunctioning stomas in low anterior resection for rectal cancer. Br J Surg.

[31] Koperna T (2003). Cost-effectiveness of defunctioning stomas in low anterior resections for rectal cancer: a call for benchmarking. Arch Surg.

[32] Ashraf SQ, Burns EM, Jani A, Altman S, Young JD, Cunningham C (2013). The economic impact of anastomotic leakage after anterior resections in English NHS hospitals: are we adequately remunerating them?. Color Dis.

