# Supplementary material for: The economic impact of anastomotic leak after colorectal cancer surgery
Source: Health Econ Rev. 2023 Feb 16;13:12. doi: 10.1186/s13561-023-00425-y (PMC9933261; doi:10.1186/s13561-023-00425-y)
Supplement: Supplementary file 1 — Additional file 1: Table S1. Consumption of resources for the diagnosis of anastomotic leak (AL). Table S2. Consumption of resources for the treatment of AL in GROUP 1 (patients with CC and AL) vs. patients without AL. Table S3. Consumption of resources for the treatment of AL in GROUP 2 (patients with RC and AL) vs. patients without AL. Table S4. Consumption of resources for the treatment of AL in GROUP 3 (patients with RC and with a protective stoma suffering from AL) vs. patients without AL. [file 13561_2023_425_MOESM1_ESM.docx]

S1: Consumption of resources for the diagnosis of anastomotic leak (AL).

| **CONSULTATIONS** | **With AL in colon cancer** | | **With AL in rectal cancer** | |
| --- | --- | --- | --- | --- |
|  | **N** | **% of patients** | **N** | **% of patients** |
| **Post-operative visits (inpatient)** | 3 in the day | 100% | 3 in the day | 100% |
| **Radiologist consultation** | 0 | 100% | 1 | 100% |
| **Emergency visit** | 1 | 20% | 1 | 20% |
| **DIAGNOSTIC TESTS** | **With AL in colon cancer** | | **With AL in rectal cancer** | |
|  | **N** | **% of patients** | **N** | **% of patients** |
| **C-reactive protein (CRP)/ procalcitonin** | 1 | 100% | 1 | 100% |
| **computed tomography (CT)** | 1 | 60% | 1 | 40% |
| **CT-enema** | 1 | 40% | 1 | 60% |

S2: Consumption of resources for the treatment of AL in GROUP 1 (patients with CC and AL) vs. patients without AL.

|  | **Incremental resources GROUP 1 (patients with CC)** | | | | | | |
| --- | --- | --- | --- | --- | --- | --- | --- |
| HOSPITAL STAY | **AL type B** | | | **AL type C** | | **AL type C + STOMA** | |
|  | **days** | **% of patients** | **days** | | **% of patients** | **days** | **% of patients** |
| **Inpatient stay** | 15 | 90% | 26 | | 40% | 26 | 40% |
| **Intensive Care Unit (ICU)** | 3 | 10% | 8 | | 60% | 8 | 60% |
| **Emergency visit** | 1 | 15% | 1 | | 5% | 1 | 5% |
| **CONSULTATIONS** | **N** | **% of patients** | **N** | | **% of patients** | **N** | **% of patients** |
| **Post-operative visits (inpatient)** | 15 | 100% | 30 | | 100% | 30 | 100% |
| **Surgeon** | 7 | 100% | 8 | | 100% | 8 | 100% |
| Radiologist | 4 | 100% | 7 | | 100% | 7 | 100% |
| **Stomatherapist** | - | - | - | | - | 10 | 100% |
| **Nutritionist/internist/rehabilitator** | 6 | 80% | 14 | | 100% | 14 | 100% |
| **TEST** | | | | | | | |
| **C-reactive protein (CRP)/ procalcitonin** | 3 | 100% | 5 | | 100% | 5 | 100% |
| **Blood test** | 10 | 100% | 16 | | 100% | 16 | 100% |
| **computed tomography (CT)** | 5 | 10% | 5 | | 80% | 5 | 80% |
|  | 4 | 90% | 4 | | 20% | 4 | 20% |
| **CT enema or opaque enema** | 0 | 100% | 0 | | 100% | 1 | 50% |
| **Colonoscopy** | 1 | 10% | 1 | | 10% | 1 | 10% |
| **INTERVENTIONS** | | | | | | | |
| **Reintervention** | - | 100% | 1 | | 60% | 1 | 40% |
|  |  | 100% | 2 | | 30% | 2 | 40% |
|  |  | 100% | 3 | | 10% | 3 | 20% |
| **Stoma closure** | - | - | - | | - | 1 | 60% |
| **DRUGS** | | | | | | | |
| **Antibiotics (Imipenem o piperacillin)** | 15 | 100% | 20 | | 100% | 20 | 100% |
| **OTHER RECOURSES** | | | | | | | |
| **Parenteral nutrition** | 7 | 50% | 14 | | 75% | 14 | 75% |
| **Enteral nutrition** | 5 | 50% | 10 | | 75% | 10 | 75% |
| **percutaneous drainage** | 0 | 60% | 1 | | 50% | 1 | 50% |
|  | 1 | 30% |  |  |  |  |  |
|  | 2 | 10% |  |  |  |  |  |
| **Stoma material** | - | - | - | | - | 1 | 100% |

S3: Consumption of resources for the treatment of AL in GROUP 2 (patients with RC and AL) vs. patients without AL.

|  | **Incremental resources GROUP 2 (patients with RC without a protective stoma)** | | | | | | | |
| --- | --- | --- | --- | --- | --- | --- | --- | --- |
| **HOSPITAL STAY** | **AL type A** | | **AL type B** | | **AL type C** | | **AL type C + STOMA** | |
|  | **days** | **% of patients** | **days** | **% of patients** | **days** | **% of patients** | **days** | **% of patients** |
| **Inpatient stay** | 15 | 100% | 22 | 90% | 28 | 40% | 28 | 40% |
| **Intensive Care Unit (ICU)** | 0 | 100% | 3 | 10% | 8 | 60% | 8 | 60% |
| **Emergency visit** | 1 | 10% | 1 | 15% | 1 | 5% | 1 | 5% |
| **CONSULTATIONS** | **N** | **% of patients** | **N** | **% of patients** | **N** | **% of patients** | **N** | **% of patients** |
| **Post-operative visits (inpatient)** | 15 | 100% | 22 | 100% | 32 | 100% | 32 | 100% |
| **Surgeon** | 6 | 100% | 8 | 100% | 8 | 100% | 8 | 100% |
| **Radiologist** | 3 | 100% | 5 | 100% | 7 | 100% | 7 | 100% |
| **Stomatherapist** | - | - | - | - | - | - | 10 | 100% |
| **Nutritionist/internist/rehabilitator** | 1 | 60% | 7 | 100% | 14 | 100% | 14 | 100% |
| **TEST** | | | | | | | | |
| **C-reactive protein (CRP)/ procalcitonin** | 3 | 100% | 3 | 100% | 5 | 100% | 5 | 100% |
| **Blood test** | 9 | 100% | 11 | 100% | 16 | 100% | 16 | 100% |
| **computed tomography (CT)** | 2 | 80% | 4 | 20% | 4 | 80% | 5 | 80% |
|  | 3 | 20% | 3 | 80% | 3 | 20% | 4 | 20% |
| **CT-enema or opaque enema** | 1 | 50% | 1 | 50% | 1 | 50% | 1 | 80% |
| **Rectoscopy/ colonoscopy (diagnostic)** | - | 100% | 1 | 10% | 1 | 10% | 1 | 20% |
| **INTERVENTIONS** | | | | | | | | |
| **Reintervention** | - | 100% | - | 100% | 1 | 60% | 1 | 40% |
|  |  | 100% |  | 100% | 2 | 30% | 2 | 40% |
|  |  | 100% |  | 100% | 3 | 10% | 3 | 20% |
| **Stoma closure** | - | - | - | - | - | - | 1 | 60% |
| **DRUGS** | | | | | | | | |
| **Antibiotics (Imipenem o piperacillin)** | 0 | 100% | 20 | 100% | 20 | 100% | 20 | 100% |
| **OTHER RECOURSES** | | | | | | | | |
| **Parenteral nutrition** | - | 100% | 7 | 50% | 14 | 75% | 14 | 75% |
| **Enteral nutrition** | 2 | 25% | 5 | 50% | 10 | 75% | 10 | 75% |
| **percutaneous drainage** | - | 100% | 1 | 70% | 1 | 50% | 1 | 50% |
|  |  |  | 2 | 30% |  |  |  |  |
| **Stoma material** | - | - | - | - | - | - | 1 | 100% |
| **Transanal drainage (endo-sponge)** | - | 100% | 5 | 10% | - | 100% | 5 | 5% |

S4: Consumption of resources for the treatment of AL in GROUP 3 (patients with RC and with a protective stoma suffering from AL) vs. patients without AL.

|  | **Incremental resources GROUP 3 (patients with RC and with a protective stoma)** | | | | | | |
| --- | --- | --- | --- | --- | --- | --- | --- |
| **HOSPITAL STAY** | **AL type A** | | | **AL type B** | | **AL type C** | |
|  | **days** | **% of patients** | **days** | | **% of patients** | **days** | **% of patients** |
| **Inpatient stay** | 14 | 100% | 20 | | 90% | 25 | 80% |
| **Intensive Care Unit (ICU)** | 0 | 100% | 3 | | 10% | 8 | 20% |
| Emergency visit | 1 | 10% | 1 | | 15% | 1 | 5% |
| **CONSULTATIONS** | **N** | **% of patients** | **N** | | **% of patients** | N | **% of patients** |
| Post-operative visits (inpatient) | 14 | 100% | 20 | | 100% | 28 | 100% |
| **Surgeon** | 6 | 100% | 8 | | 100% | 8 | 100% |
| **Radiologist** | 3 | 100% | 5 | | 100% | 7 | 100% |
| **Stomatherapist** | 5 | 100% | 5 | | 100% | 6 | 100% |
| **Nutritionist/internist/rehabilitator** | 1 | 60% | 7 | | 100% | 14 | 100% |
| **TEST** | | | | | | | |
| **C-reactive protein (CRP)/ procalcitonin** | 3 | 100% | 3 | | 100% | 5 | 100% |
| **Blood test** | 9 | 100% | 11 | | 100% | 16 | 100% |
| **computed tomography (CT)** | 2 | 80% | 4 | | 20% | 4 | 80% |
|  | 3 | 20% | 3 | | 80% | 3 | 20% |
| **CT-enema or opaque enema** | 1 | 80% | 2 | | 100% | 2 | 100% |
| **CT-enema or opaque enema (stoma closure)** | 1 | 100% | 1 | | 100% | 1 | 100% |
| **Rectoscopy/ colonoscopy (diagnostic)** | 0 | 100% | 1 | | 10% | 1 | 10% |
| **INTERVENTIONS** | | | | | | | |
| **Reintervention** | - | 100% | - | | 100% | 1 | 85% |
|  |  |  |  |  |  | 2 | 10% |
|  |  |  |  |  |  | 3 | 5% |
| **Stoma closure *(only AL type C + stoma)*** | 1 | 70% | 1 | | 50% | 1 | 50% |
| **DRUGS** | | | | | | | |
| **Antibiotics (Imipenem o piperacillin)** | 0 | 100% | 20 | | 100% | 20 | 100% |
| **OTHER RECOURSES** | | | | | | | |
| **Parenteral nutrition** | 0 | 100% | 5 | | 20% | 10 | 40% |
| **Enteral nutrition** | 2 | 25% | 5 | | 50% | 10 | 75% |
| **percutaneous drainage** | 0 | 100% | 1 | | 70% | 1 | 50% |
|  |  |  | 2 | | 30% |  |  |
| **Stoma material** | 1 | 100% | 1 | | 100% | 1 | 100% |
| **Transanal drainage (endo-sponge)** | 0 | 100% | 5 | | 20% | 5 | 10% |
