# Supplementary material for: The economic impact of anastomotic leak after colorectal cancer surgery
Source: Health Econ Rev. 2023 Feb 16;13:12. doi: 10.1186/s13561-023-00425-y (PMC9933261; doi:10.1186/s13561-023-00425-y)
Supplement: Supplementary file 2 — Additional file 2: Table S5. Unit cost of resources for the diagnosis and treatment of AL. [file 13561_2023_425_MOESM2_ESM.docx]

S5: Unit cost of resources for the diagnosis and treatment of AL.

| Recourse | Unitary cost (€, 2021) | Reference |
| --- | --- | --- |
| HOSPITAL STAY | |  |
| Inpatient stay (per day) | €663.25 | eSalud, 2021 [1] |
| Stay in the Intensive Care Unit (ICU) per day | €2,250.35 |  |
| Emergency visit | €177.37 |  |
| CONSULTATIONS | |  |
| surgeon | €115.71 | eSalud, 2021 [1] |
| radiologist | €88.91 |  |
| Stomatherapist | €261.32 |  |
| Nutritionist/ internist/ rehabilitator | €132.64 |  |
| Post-operative visit | €52.90 |  |
| TEST | |  |
| Blood test | €78.39 | eSalud, 2021 [1] |
| Colonoscopy | €243.07 |  |
| C-reactive protein (CRP) + procalcitonin | €61.64 |  |
| Rectoscopy | €126.44 |  |
| CT | €383.60 |  |
| CT enema | €441.80 |  |
| INTERVENTIONS | |  |
| Reintervention* | €6,445.45 | Mar J. et al 2017 [2] |
| Stoma closure** | €1,968.19 | eSalud, 2021 [1] |
| DRUGS | |  |
| Antibiotics (drug + administration) | €182.03 | eSalud, 2021 [1] |
| OTHER RECOURSES | |  |
| Percutaneous drainage | €231.75 | eSalud, 2021 [1] |
| Drenaje transanal | €157.00 |  |
| Stoma material*** | €102.26 | García-Goñi M, 2019 [3] |
| Enteral nutrition | €463.66 | eSalud, 2021 [1] |
| Parenteral nutrition | €157.00 |  |

*Average cost DRG right hemicolectomy, left hemicolectomy, sigmodectomy, colectomy, other colonic excision, anterior rectal resection, abdominoperineal amputation, and other rectal excision. ** procedure cost €46.52-Closure of large intestine stoma. ***Monthly cost obtained from García-Goñi M, 2019 [3].

**References**

1. Oblikue - Base de conocimiento de costes y precios del sector sanitario [Internet]. [cited 2021 Jul 1]. Available from: http://esalud.oblikue.com/

2. Mar J, Errasti J, Soto-Gordoa M, Mar-Barrutia G, Martinez-Llorente JM, Domínguez S, et al. The cost of colorectal cancer according to the TNM stage. Cir Esp. 2017;95:89–96.

3. García-Goñi M. Specializing Nurses as An Indirect Education Program for Stoma Patients. Int J Environ Res Public Health. 2019;16:E2272.
